# Supplementary material for: Origins of direction selectivity in the primate retina
Source: Nat Commun. 2022 May 23;13:2862. doi: 10.1038/s41467-022-30405-5 (PMC9126974; doi:10.1038/s41467-022-30405-5)
Supplement: Supplementary file 1 — Supplementary Information [file 41467_2022_30405_MOESM1_ESM.pdf]

Supplementary Information

Origins of direction selectivity in the primate retina

By Kim et al.

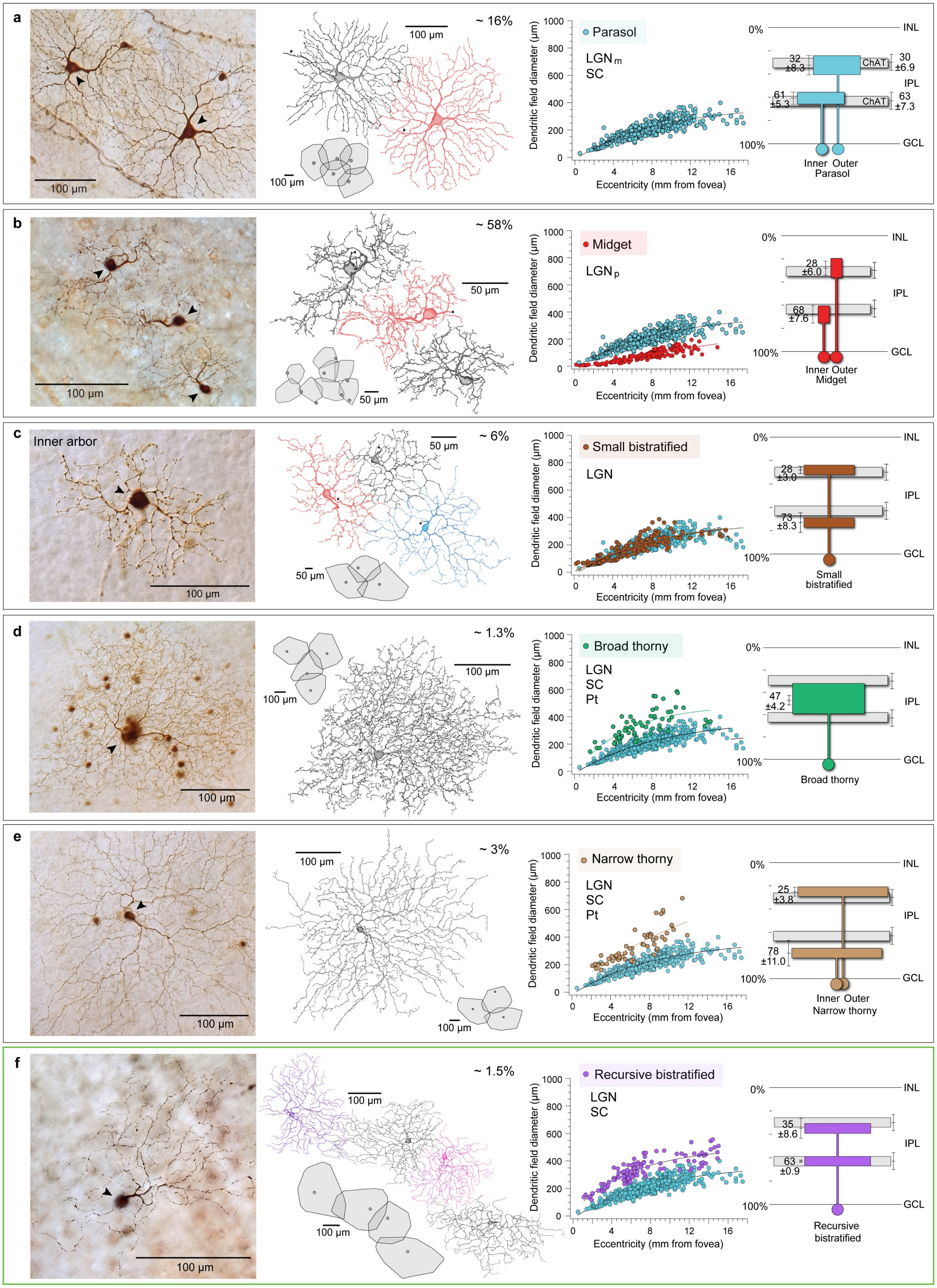
a
100 µm
~ 16%
100 µm
100 µm
Parasol
LGN m
SC
Dendritic field diameter (µm)
Eccentricity (mm from fovea)
0%
INL
32
±8.3
ChAT
30
±6.9
IPL
61
±5.3
ChAT
63
±7.3
100%
GCL
Inner Outer
Parasol
b
100 µm
~ 58%
50 µm
50 µm
Midget
LGN p
0%
INL
28
±6.0
IPL
68
±7.6
100%
GCL
Inner Outer
Midget
c
Inner arbor
100 µm
50 µm
~ 6%
50 µm
Small bistratified
LGN
0%
INL
28
±3.0
IPL
73
±8.3
100%
GCL
Small
bistratified
d
100 µm
100 µm
~ 1.3%
100 µm
Broad thorny
LGN
SC
Pt
0%
INL
47
±4.2
IPL
100%
GCL
Broad thorny
e
100 µm
100 µm
~ 3%
100 µm
Narrow thorny
LGN
SC
Pt
0%
INL
25
±3.8
IPL
78
±11.0
100%
GCL
Inner Outer
Narrow thorny
f
100 µm
~ 1.5%
100 µm
100 µm
Recursive bistratified
LGN
SC
0%
INL
35
±8.6
IPL
63
±0.9
100%
GCL
Recursive
bistratified

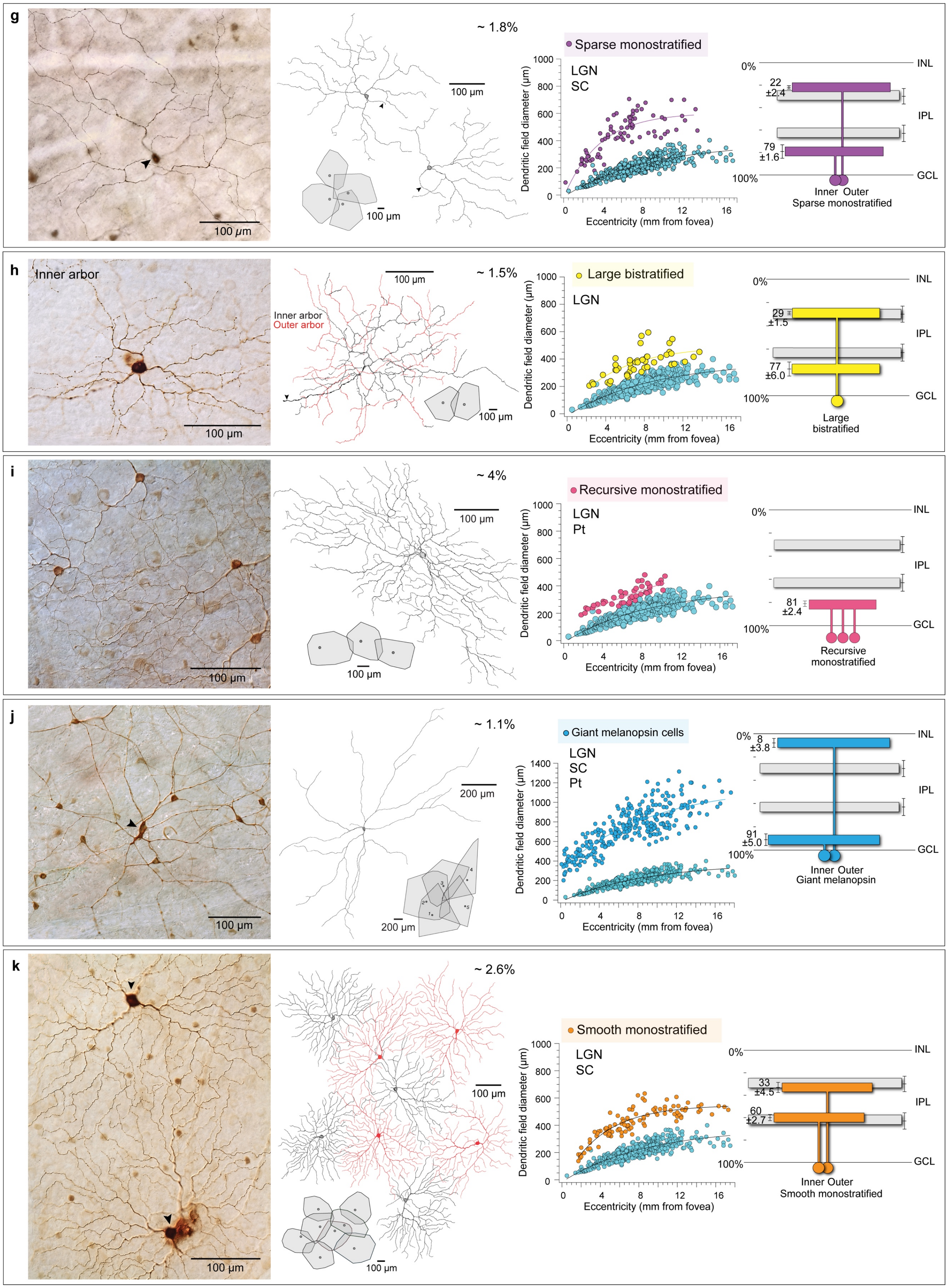

g
100 μm
~ 1.8%
100 μm
100 μm
Sparse monostratified
LGN
SC
Dendritic field diameter (μm)
Eccentricity (mm from fovea)
0%
INL
22
±2.4
IPL
79
±1.6
100%
GCL
Inner Outer
Sparse monostratified
h
Inner arbor
100 μm
100 μm
~ 1.5%
Inner arbor
Outer arbor
100 μm
Large bistratified
LGN
Dendritic field diameter (μm)
Eccentricity (mm from fovea)
0%
INL
29
±1.5
IPL
77
±6.0
100%
GCL
Large
bistratified
i
100 μm
~ 4%
100 μm
100 μm
Recursive monostratified
LGN
Pt
Dendritic field diameter (μm)
Eccentricity (mm from fovea)
0%
INL
IPL
81
±2.4
100%
GCL
Recursive
monostratified
j
100 μm
~ 1.1%
200 μm
200 μm
Giant melanopsin cells
LGN
SC
Pt
Dendritic field diameter (μm)
Eccentricity (mm from fovea)
0%
8
±3.8
INL
IPL
91
±5.0
100%
GCL
Inner Outer
Giant melanopsin
k
100 μm
~ 2.6%
100 μm
100 μm
Smooth monostratified
LGN
SC
Dendritic field diameter (μm)
Eccentricity (mm from fovea)
0%
INL
33
±4.5
IPL
60
±2.7
100%
GCL
Inner Outer
Smooth monostratified

Supplementary Figure 1. Morphology, spatial densities, and stratification depth of 19 proposed ganglion-cell types in the macaque monkey retina. A near complete atlas of the ganglion-cell types reveals recursive bistratified ganglion cells are present as a single low-density population. **a**, Parasol cells comprise ~16% of total ganglion cells (coverage: inner cells 1.9; outer cells 1.6) and project to the lateral geniculate nucleus (LGN), superior colliculus (SC) and pretectum (Pt). Left, photomicrograph of two photo-stained cells tracer labeled from injections in the SC (arrowheads, cell bodies; HRP histochemistry). Center, tracings of the adjacent dendritic trees of the two cells shown on the left (arrowheads, axons; inset at bottom, Outlines of the overlapping dendritic fields of 5 neighboring outer parasol cells ~6 mm from the fovea. Right, dendritic field diameter plotted against retinal eccentricity (n = 438 cells; mean ± sd = 206 ± 65 μm diam; range = 30.5 - 400 μm) and mean dendritic stratification measured in wholemount retina and plotted as percentage depth in the IPL (inner cells, n = 36; outer cells, n = 20) and relative to choline acetyl transferase (ChAT) immunostained bands (see Supplementary Fig. 3 and Methods). Conventions in all subsequent panels follow panel **a**. **b,** Midget ganglion cells include inner (ON-center) and outer (OFF-center) stratifying types that comprise ~58% of total ganglion cells (coverage: inner cells 1.1; outer cells 1.1) and project to the LGN. Left, photomicrograph of three photo-stained inner midget cells (HRP histochemistry). Center, tracings of the cells shown in a; shows dendritic coverage (arrows indicate axons); inset at bottom, outlines of the overlapping dendritic fields of eight neighboring inner midget cells. Right, dendritic field diameter plotted against retinal eccentricity (red, n = 181; mean ± sd = 84 ± 41; range = 4.9 - 195.5 μm diam); in this and all the following panels the dendritic filed diameters of

parasol cells (blue) are shown for comparison). Mean dendritic stratification measured relative to ChAT bands (Supplementary Fig. 3b). **c**, Small bistratified cells comprise ~6% of total ganglion cells (coverage = 1.6) and project to the LGN. Left, small bistratified cell intracellular Neurobiotin fill (HRP histochemistry); focus on the inner dendritic arbor. Center, tracings of three additional photostained cells showing dendritric coverage (HRP histochemistry); arrowheads indicate axons. Inset at bottom, outlines of the overlapping dendritic fields of the three cells. Right, dendritic field diameter plotted as a function of eccentricity (orange; n = 191; mean ± sd = 178 ± 76; range = 52 - 388 µm); mean dendritic stratification measured relative to ChAT bands (Supplementary Fig. 3c). **d**, Broad thorny cells form a single population that comprises ~1.5% of total ganglion cells and project to the LGN, SC and pretectum. Left, photomicrograph of a broad thorny cell at 6.8 mm eccentricity in inferior retina, intracellularly injected with Neurobiotin and processed for HRP histochemistry. The cell showed tracer-coupling to a population of small-bodied amacrine cells. Center, tracing of the same cell. Arrowhead indicates axon; inset, outlines of the overlapping dendritic fields of 4 neighboring broad thorny cells retrogradely photostained from the SC (coverage = 1.2). Right, dendritic field diameter (green) plotted as a function of eccentricity (mean dendritic field diam ± sd = 331 ± 102 µm; range = 146 - 586 µm; n = 67). Dendritic stratification measured relative to Chat bands (Supplementary Fig. 3d). **e**, Narrow thorny cells form inner and outer stratifying populations that comprise ~3% of total ganglion cells and project to the LGN, SC and pretectum. Left, photomicrograph of an outer narrow thorny cell (intracellular Neurobiotin fill; HRP histochemistry; arrow indicates the cell body). Center, tracing of the same cell shown in A. Arrowhead indicates axon; inset, outlines of the

overlapping dendritic fields of 3 neighboring outer narrow thorny cells retrogradely labeled from tracer injections in the SC (coverage: inner cells 1.0, outer cells 1.0) Right, dendritic field diameter (gold) plotted as a function of eccentricity; mean dendritic field diameter ± sd = 331 ± 119 µm; range = 163 - 682 µm; n = 59. Mean dendritic stratification measured relative to ChAT bands. **f**, Recursive bistratified cells comprise ~1.5% of total ganglion cells (coverage = 1.3) and project to the LGN and SC. Photomicrograph of a recursive bistratified cell intracellularly injected with Neurobiotin (HRP histochemistry). The cell shows tracer coupling to multiple amacrine-cell populations. Center, tracings of four cells photo-stained from the SC (this mosaic is also shown, rotated in Fig. 2a); inset, outlines of the overlapping dendritic fields of the same four cells. Right, dendritic field diameter plotted as a function of eccentricity (n = 122; mean ± sd = 327 ± 93; range = 117 - 557µm). Mean dendritic stratification measured relative to parasol stratification in wholemount retina and plotted as percentage depth in the IPL (recursive bistratified/parasol cell pairs, n = 3; see Methods). **g**, Sparse monostratified cells form inner and outer stratifying populations that comprise 1.8 of total ganglion cells and project to the LGN and the SC. Left, photomicrograph of an outer sparse monostratified cell photostained from the SC (HRP histochemistry; (arrowhead, cell body). Center, tracings of two neighboring inner sparse monostratified cells; inset; outlines of the overlapping dendritic fields of four neighboring outer sparse monostratified cells (coverage: inner cells 1.5, outer cells 1.5). Right, dendritic field diameter (purple) plotted as a function of eccentricity (mean dendritic field diameter ± sd = 447 ± 143 µm; range = 93 - 706 µm; n = 78). Mean dendritic stratification measured relative to starburst amacrine-cell dendrites (ChAT bands) in wholemount retina and

plotted as percentage depth in the IPL (inner cells, n = 1; outer cells, n = 3). **h**, Large bistratified cells make up 1.5% of total ganglion cells (coverage: ~ 2) and project to the LGN. Left, photomicrograph of a large bistratified cell, plane of focus, inner dendritic arbor (Neurobiotin fill; HRP histochemistry). Center, tracing of the same cell shown in photomicrograph (outer dendritic arbor is shown in red, arrowhead indicates axon); inset, outlines of the dendritic fields of two neighboring large bistratified cells with overlapping dendritic fields. Right, dendritic field diameter plotted as a function of eccentricity (n = 46, mean ± sd = 365 ± 92, range = 206 - 595 μm diam). Mean dendritic stratification relative to Chat bands. **i**, Recursive monostratified cells comprise 4.2% (3 populations; single population: ~1.3%) of total ganglion cells (coverage: single population 1.2; 3 populations 3.5) and project to the LGN and pretectum. Left, photomicrograph of photostained cells from pretectum tracer injections (HRP histochemistry). Center, tracing of a recursive monostratified cell (arrowhead, axon); inset, outlines of the overlapping dendritic fields of 3 neighboring recursive monostratified cells. Right, dendritic field diameter plotted as a function of eccentricity (n = 39, mean ± sd = 323 ± 77, range = 188 - 480 μm diam). Mean dendritic stratification measured relative to ChAT bands. **j**, Giant melanopsin cells form inner and outer stratifying populations that comprise ~1.1% of total ganglion cells and project to the LGN, SC and pretectum. Left, photomicrograph of a giant melanopsin cell (arrowhead) intracellularly injected with Neurobiotin (HRP histochemistry). The cell shows Neurobiotin tracer-coupling to several populations of amacrine cells (smaller cell bodies surrounding the ganglion cell). Tracing of an inner giant melanopsin immunolabeled with human Melanopsin antibody and processed for HRP histochemistry. Bottom, outlines of

the overlapping dendritic fields of 5 neighboring inner Melanopsin cells (coverage: inner cells 1.5, outer cells 2.0). Right, dendritic field diameter (red circles) plotted as a function of eccentricity (dark blue, mean dendritic field diameter ± SD = 740 ± 219 μm; range = 202 - 1316 μm; n = 276). Mean dendritic stratification plotted relative to the Chat bands (inner giant cell: n = 2; outer giant cell: n = 2). **k**, Smooth monostratified cells comprise 2.6% of total ganglion cells (coverage: inner cells 1.4; outer cells 1.4) and project to the LGN, SC and pretectum. Left, photomicrograph of two smooth cells (Neurobiotn fill; HRP histochemistry). The smooth cells are tracer-coupled to a population of small-bodied amacrine cells. Tracings of eight inner smooth cells, photostained after SC tracer injection; inset, outlines of the overlapping dendritic fields of the eight cells. Right, dendritic field diameter plotted as a function of eccentricity (n = 102, mean ± sd = 419 ±110, range = 138 - 634 μm diam). Mean dendritic stratification measured relative to Chat bands. For all panels: GCL, ganglion-cell layer; IPL, inner plexiform layer; INL, inner nuclear layer.

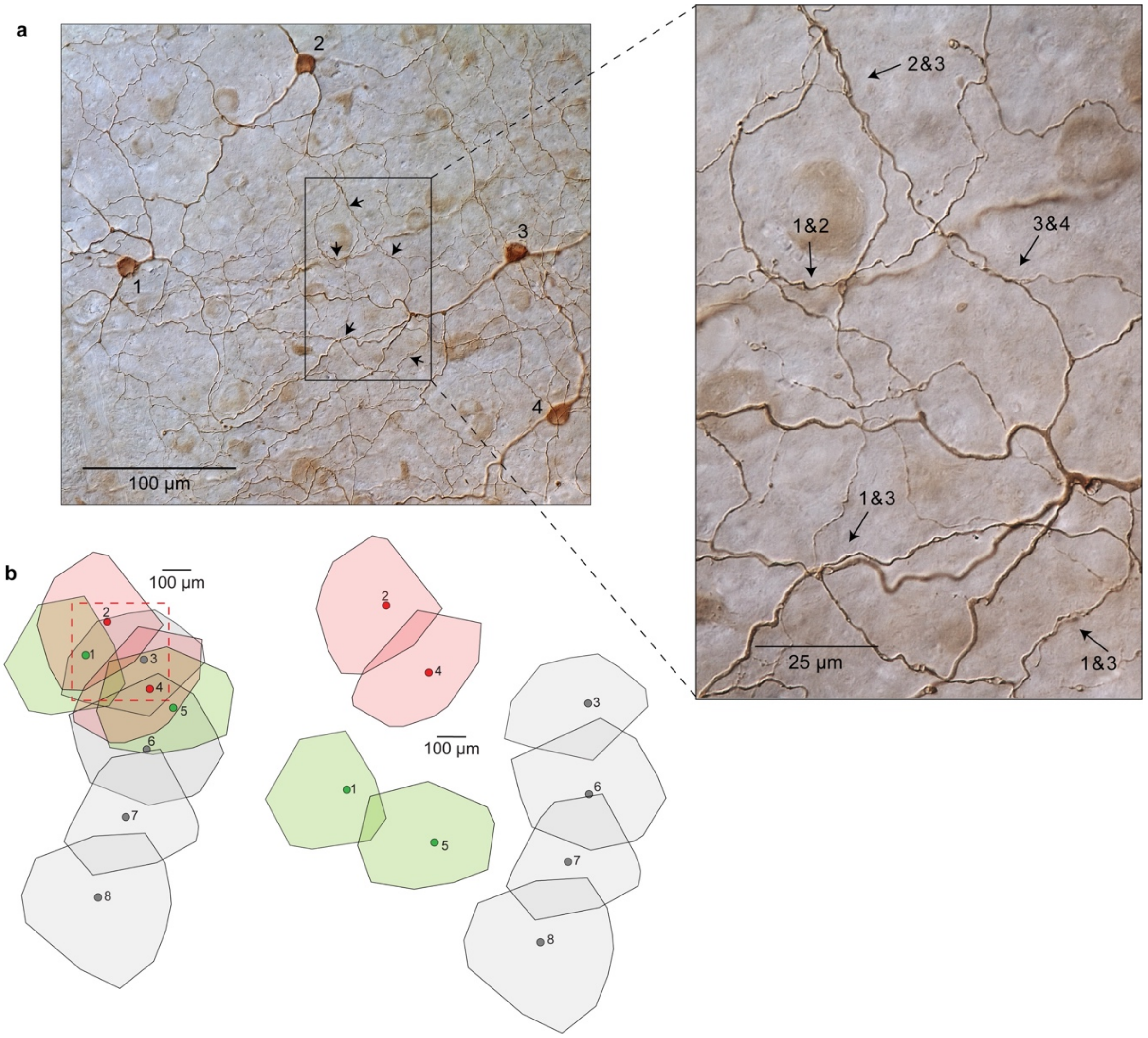

Supplementary Figure 2. Recursive monostratified cells show fasciculating dendrites and at least 3 multiple overlapping cellular mosaics. a, Photomicrograph of a patch of photo-stained cells tracer labeled from injections in the pretectum and reacted for HRP histochemistry. The dendrites of the cell bodies labeled 1 thru 4 and other nearby cells (cell bodies outside of image) form a dense plexus of co-fasciculated processes. Right, magnified view of boxed area in **a**. Arrows indicate 5 examples of co-fasciculation. Numbers correspond to the dendrites' cells of origin as shown in **a**. **b,** The highly overlapping and cofasiculating dendrites of cells 1-3 appear to be components of three separate ganglion-cell mosaics (red dotted line boxed area corresponds to

location of image shown in **a**) illustrated by the tiles shown in pink (cells 2, 4), gray (cells, 3,6-8) and green (cells 1, 5), traced around additional neighboring dendritic trees with minimal overlap.

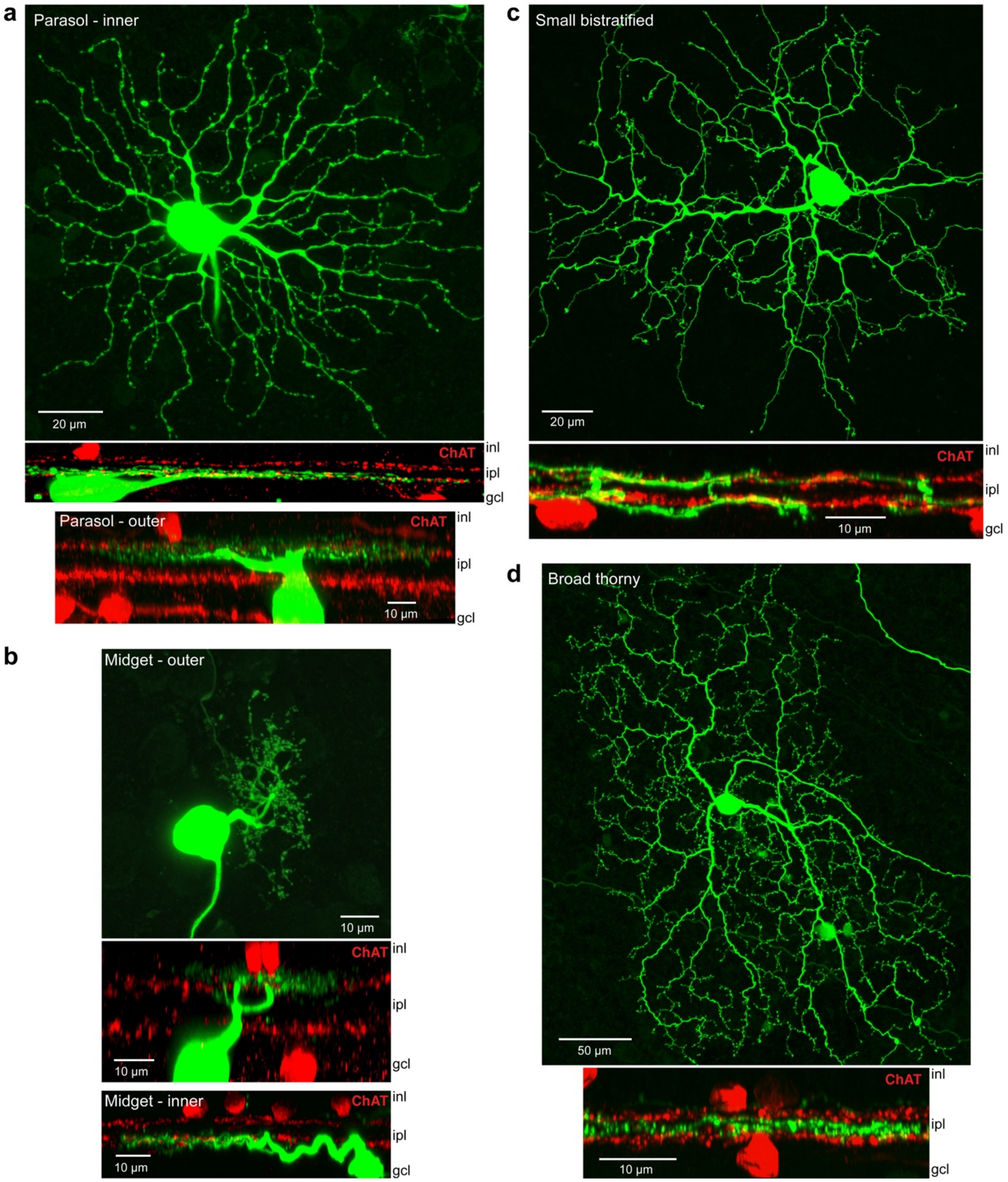

Supplementary Figure 3. Stratification levels of distinct ganglion-cell types relative to ChAT-labeled starburst amacrine cells. a-d, Upper panels show maximum intensity projections from confocal image stacks of ganglion cells injected

with Neurobiotin in whole-mounted macaque retina. Neurobiotin was visualized by dye-coupled streptavidin and the specimens were double-labeled with antibodies against choline acetyl transferase (ChAT). Lower panels show z-reconstructions (vertical views) from different regions of the ganglion cells' dendritic fields together with ChAT staining. **a,** Dendrites of inner parasol cells (green) costratify with the inner ChAT band (red). Dendrites of an outer parasol cell (bottom panel) show an inversed pattern of stratification levels relative to the outer ChAT band. **b,** The outer (upper and middle panel) and inner (bottom panel) midget cells' dendritic ramifications overlap with the ChAT bands, but are broad and also extend deep (inner midgets) or superficial (outer midgets) to the ChAT bands. **c,** Inner dendrites of a small bistratified cell stratify within a wide region largely deep to the inner ChAT band. The outer dendritic tier ramifies largely at the level of the outer ChAT band. **d**, The dendritic ramifications of a broad thorny cell occupy the central third of the IPL in space between the outer and inner ChAT bands.

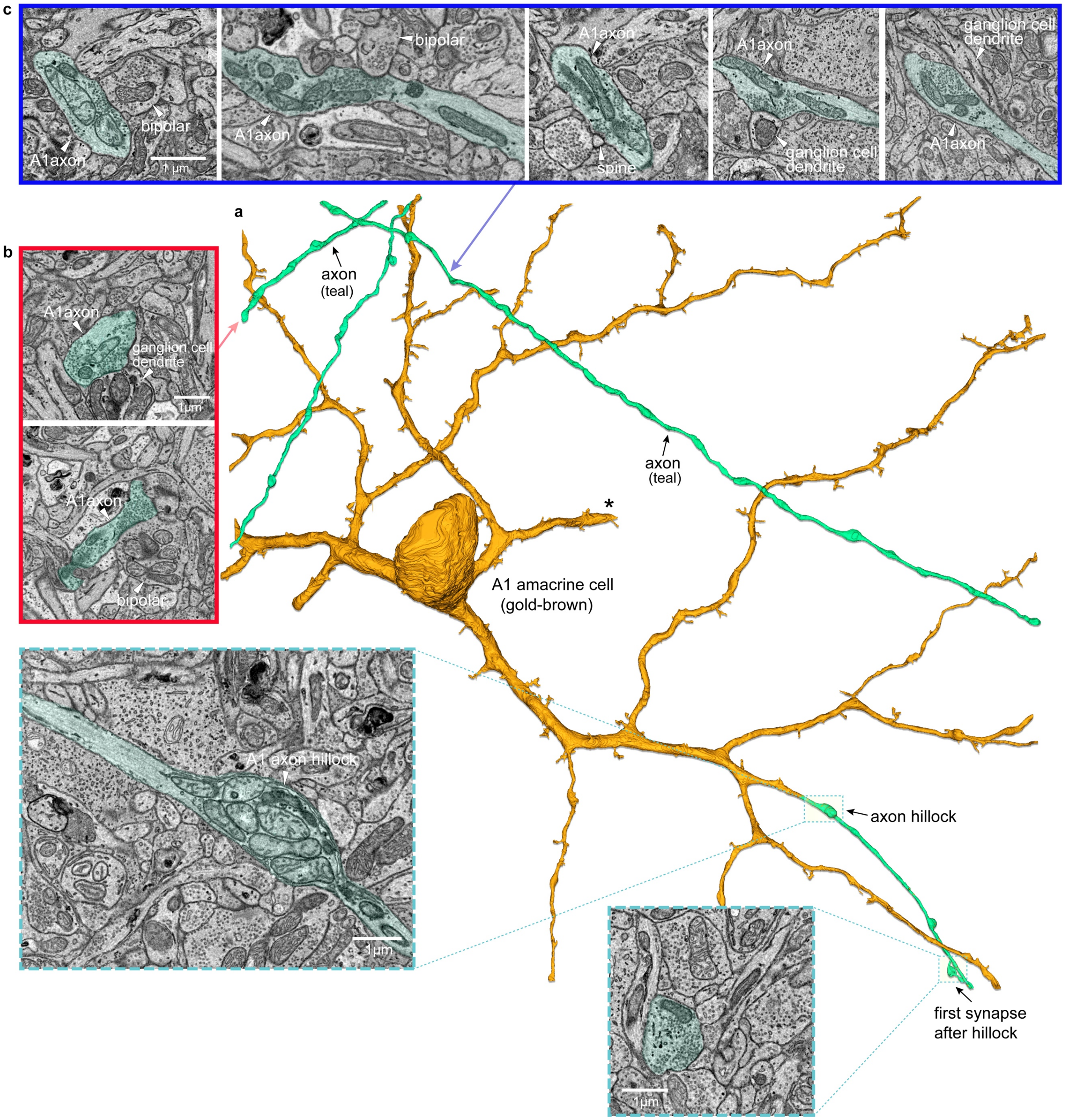

Supplementary Figure 4. Origin of A1 axon at an axon hillock like structure and the presynaptic nature of the A1 axonal processes. a, A1 cell partial reconstruction (gold; see also Fig. 5, main text) illustrating the origin of one axon from a thick proximal dendrite (teal, boxed area, axon hillock). Inset, boxed area zoomed at lower left; at its point of origin the axon swells and is densely packed with mitochondria; the axon shows

a constant diameter and lacks all synaptic input as it extends distally; the first synapse appears distal to the axon hillock (boxed area, zoomed inset; first synapse after axon hillock). Axonal processes co-stratify with the dendritic tree and are numerous; two additional examples are shown; these processes do not receive synaptic input and make synaptic output primarily to cone bipolar axon terminals (8 axon segments; 73/127 synapses; 57.5 % of total) and to a lesser extent with presumed ganglion-cell dendrites and dendritic spines (36/127 synapses, 28% of total; 18 additional synapses, 14%, either contacted other amacrine cell or unidentified processes). **b**, Red boxed panels show synapses on a ganglion-cell dendrite and a bipolar axon terminal from shorter axon segment (red arrow). **c**, Blue boxed panels show examples of synaptic output from points along longer axon segment (blue arrow) onto both bipolar axon terminals and presumed ganglion-cell dendrites. All postsynaptic bipolar-cell axon terminals were identified by ribbon synapse content and ganglion-cell dendrites were inferred by reconstruction and lack of synaptic output.

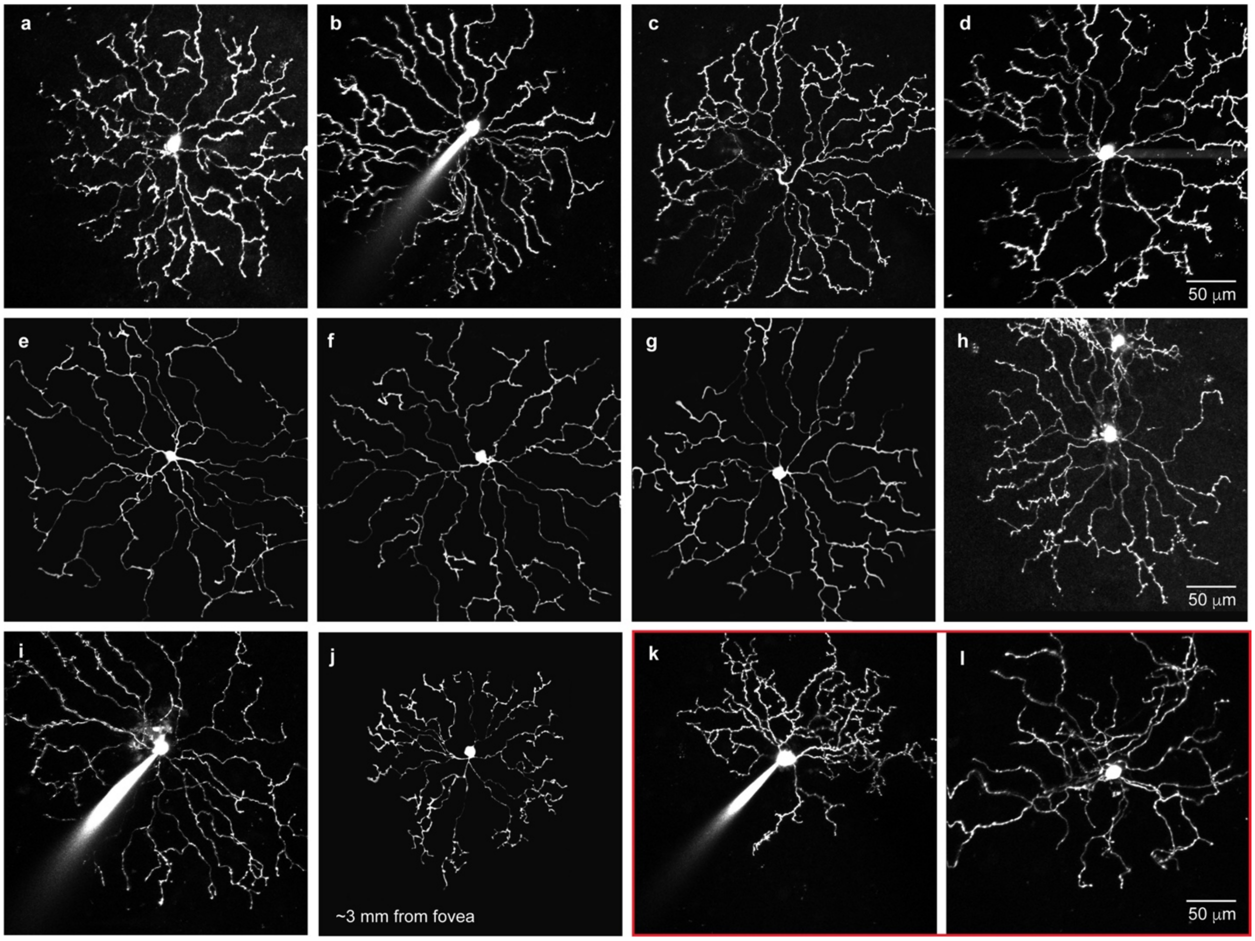

Supplementary Figure 5. Distinctive dendritic morphology of inner starburst amacrine cells in macaque monkey retina. a-j, Panels illustrate starburst cell morphological variability and the relative constancy of dendritic tree diameter across much of the peripheral retina (6-10 mm retinal eccentricity; OGB filled electrodes are evident in some images). All cells impaled and physiologically identified as inner, ON-center starbursts that displayed the same basic light response as illustrated in **Fig. 7**. **j**, Physiologically identified starburst cell at ~3 mm eccentricity shows a smaller dendritic tree (about 150 µm diam). **k-l**, Two examples of amacrine cells with radial symmetric dendritic morphology and a dendritic tree size similar to that of starburst cells but were identified as distinct 'non-starburst' amacrine-cell types due to multiple features of the

light response (e.g., extremely sustained ON response) that were not shared by all other starbursts.

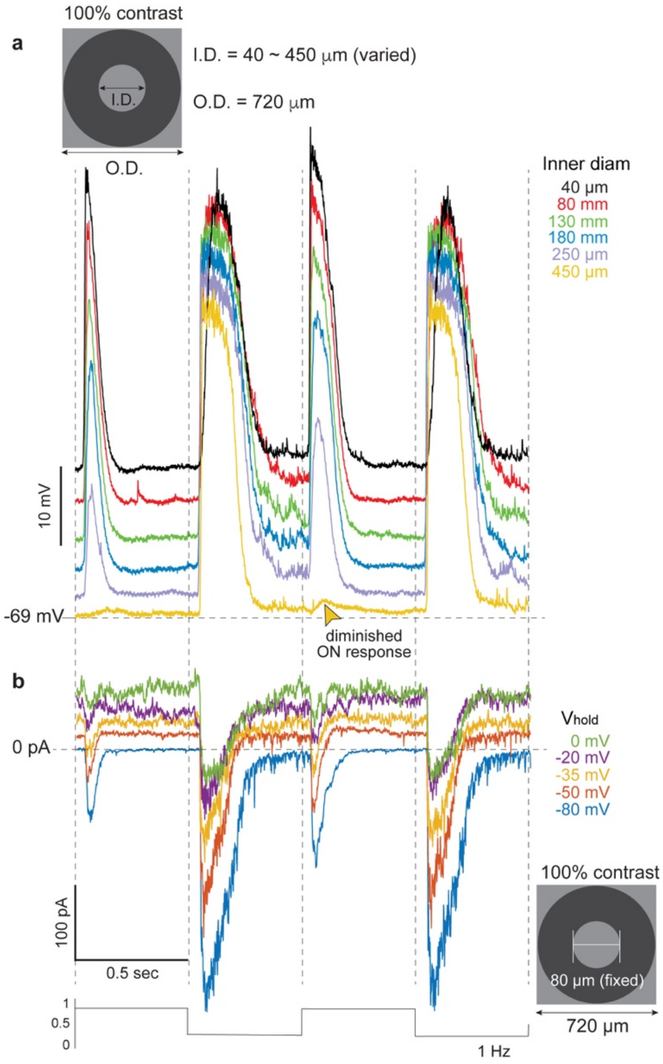

Supplementary Figure 6. Some basic features of the macaque starburst cell photopic light response. a, Whole-cell current-clamp recordings of macaque ON-center starburst cells show that these cells receive a strong excitatory input at light OFF. Top, inset, an annular stimulus was used to measure receptive-field spatial structure in which annulus inner diam (I.D.) was increased from 40 to 450 µm; stimulus was square wave modulated at 1 Hz temporal frequency. When the annulus inner diam was small (black and red traces) a large transient depolarization appears at light ONset and an equally large, more sustained depolarization appears at light OFFset. As annulus inner diam increases to encompass the receptive-field center (~300 µm) the ON component

diminishes (green thru yellow traces) but the OFF field, larger in diam than the center, remains present. Traces are shifted vertically for illustration purposes. **b**, In voltage-clamp mode, both the ON and OFF responses (inset at bottom right; annulus inner diam is fixed at 80 μm for all traces) appear as large, inward excitatory synaptic currents at negative holding potentials (V_{hold}, -80 to 0 mV, color coded). The ON component approaches equilibrium at the cation reversal potential (0 mV hold, green trace), but a small OFF inward current remains present.

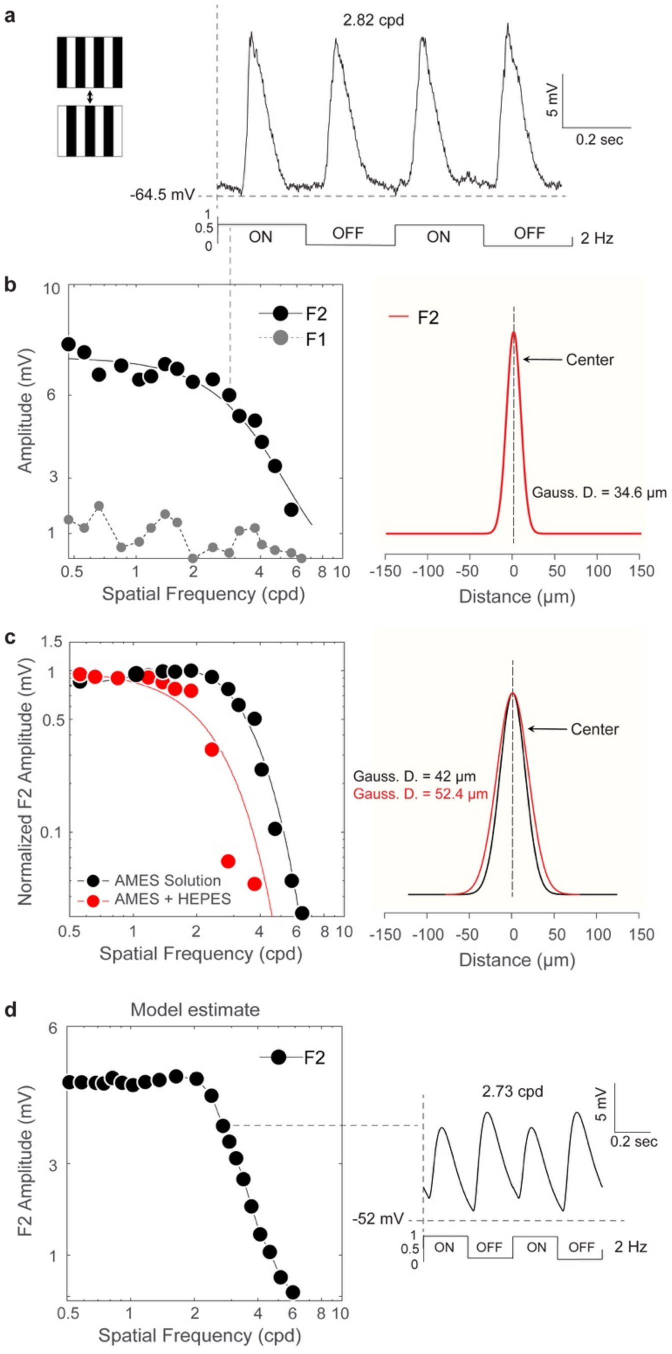

Supplementary Figure 7. Characterization of small excitatory non-linear subunits in the starburst receptive-field center. a, Counterphase modulated square-wave grating (left, inset) (100% contrast) elicits a large somatic starburst voltage response at 4 Hz, twice the 2 Hz stimulus frequency (response to 2.8 cycles/degree (cpd) grating spatial frequency). The Fourier amplitude of the second harmonic (F2) is 5.4 mV with

little response at the fundamental stimulus frequency, F1 Fourier amplitude = 0.3 mV. **b,** Left, plot of F2 amplitude with increasing stimulus spatial frequency (black data points), the F2 response extends to high spatial frequencies; F1 values are plotted for comparison (grey data points). Right, the F2 data are well fit (red line) by a single Gaussian (diam ~ 35 μm; n = 7; 55 ± 18). **c**, Left, addition of HEPES to bath to eliminate surround response (red, HEPES, black, control) has little effect on the F2 response to counterphase modulated gratings indicating that the F2 response component reflects summation of bipolar-cell receptive-field centers. **d,** F2 response to counterphase square wave gratings is reproduced in model starburst cell; the bipolar-cell center size (Gaussian diam = 30 μm) used to produce these data was used in the starburst simulations of Fig. 9 and the bipolar-cell responses of Supplementary Fig. 8.

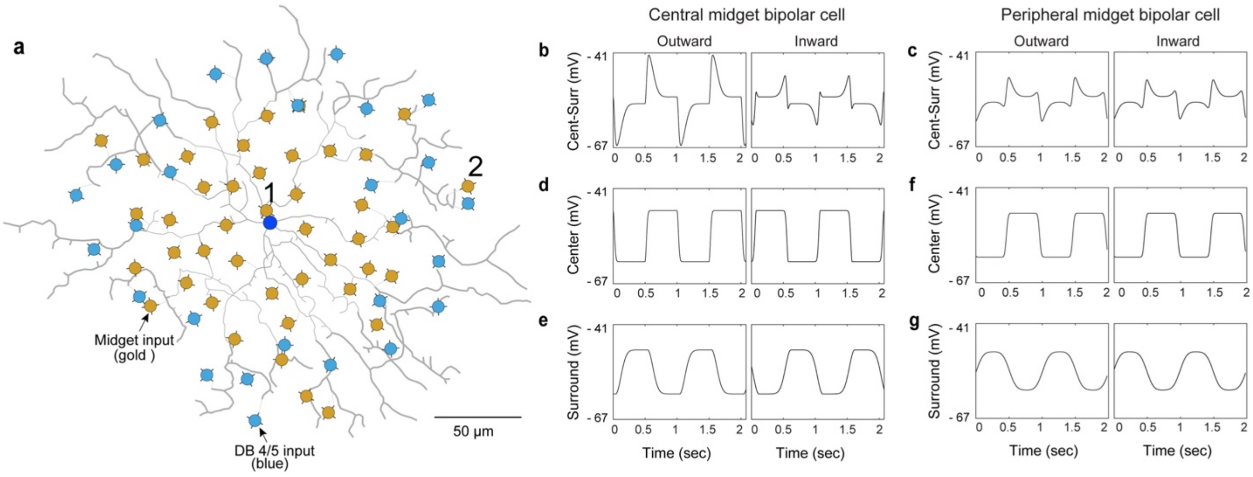

Supplementary Figure 8. Bipolar-cell responses are motion sensitive due to the delay between center and surround evoked signals. **a**. Model of starburst amacrine cell with the locations of its presynaptic bipolar cells. Gold dots, midget bipolar cells; light blue dots, DB4/5 bipolar cells. The starburst amacrine dendrites are gray and its soma is the centrally located dark blue circle. An array of midget and DB4/5 bipolar cells made synaptic connections to starburst dendrites (see Methods). To account for the differential distribution of midget and DB4/5 bipolar cells (Fig. 9) the density of midget bipolar inputs to the starburst was reduced by two-thirds beyond 65 μm from the soma and DB4/5 bipolars inserted where midgets had been removed. Modeled voltage responses of (**b**) a central midget bipolar cell (cell 1 in **a**) and (**c**) a peripheral midget bipolar cell (cell 2 in **a**) to radial moving square-wave gratings (spatial period, 500 μm, velocity 500 μm/s, "contrast" 7 mV) centered on the starburst soma in both outward and inward directions. **d** and **f**. Response of the central (**d**) and peripheral (**f**) midget bipolar cells to their respective center receptive-field components. **e** and **g**. Response of the central (**e**) and peripheral (**g**) midget bipolar cells to their respective receptive-field surround components. Responses of the bipolar cells near the starburst soma to moving radial ratings were sensitive to the direction of motion. The response to outward

motion was greater than the response to inward motion. This was not the case for peripheral bipolar cells. This difference in behavior between central and peripheral bipolar cells resulted from the time difference in engagement of the center and surround components of their receptive fields for the two conditions, outward and inward motion. By comparison, in models run with linear gratings or bars (i.e., non-radial gratings) moving across the entire starburst dendritic arbor, the bipolar-cell responses showed no sensitivity to motion (not shown). The receptive fields comprised Gaussian blurred center and surround components (center: diam 30 μm; surround: diam 120 μm, integrated weight 0.7-0.9 of center weight, delay 40 ms). When the radial grating moved outward, it engaged the delayed antagonistic surround response of a central bipolar cell after its center response. However, when the radial grating moved inward, it engaged the antagonistic surround response of a central bipolar cell before its center response, because its surround response extended spatially beyond its center response towards the inward-moving grating. Peripheral bipolar cells, including both midget and db4/db5 types, were engaged more symmetrically by the outward and inward motion. The preference of the central bipolar cells for outward-moving stimuli was transmitted to the postsynaptic starburst cell which therefore also responded to the same radial grating with a preference for outward motion as shown in Fig. 9I. These modeling results are consistent with a recent report of sensitivity to motion in bipolar cells (Strauss et al. 2021).

Summary of ganglion cell types in macaque retina

	Ganglion cell morphological type	% of total ganglion cell population*	Central injection sites	Some physiological properties
1<br>2	Midget inner<br>Midget outer	27.0<br>31.0	LGN	Achromatic, sustained<br>L vs M cone "red-green" opponent
3<br>4	Parasol inner<br>Parasol outer	8.0<br>7.8	LGN<br>Superior colliculus<br>Pretectum	Achromatic L+M cone input<br>transient
5	Small bistratified	6.1	LGN	S ON vs L+M OFF "blue-yellow" opponent
6<br>7<br>8	Recursive monostratified<br>(3 inner populations)	1.4<br>1.4<br>1.4	LGN<br>Pretectum	Possible correlate of ON direction selective type in non-primate mammals
9<br>10	Narrow thorny inner<br>Narrow thorny outer	1.5<br>1.5	LGN<br>Superior colliculus<br>Pretectum	Unknown
11<br>12	Smooth inner<br>Smooth outer	1.3<br>1.3	LGN<br>Superior colliculus	Achromatic L+M cone input
13<br>14	Sparse monostratified inner<br>Sparse monostratified outer	0.9<br>0.9	LGN<br>Superior colliculus	L+M ON; S OFF opponent<br>details unknown
15	Recursive bistratified	1.5	LGN<br>Superior colliculus	ON-OFF direction selective
16	Large bistratified	1.5	LGN	S ON opponent<br>details unknown
17	Broad thorny monostratified	1.3	LGN<br>Superior colliculus<br>Pretectum	ON-OFF, transient
18<br>19	Giant melanopsin inner<br>Giant melanopsin outer	0.5<br>0.6	LGN<br>Superior colliculus<br>Pretectum	Sustained ON response<br>S OFF; L+M ON opponent<br>Strong rod input<br>Intrinsically photosensitive

*Total ganglion cell density is from Wässle et al., 1989, for temporal retina ~ 8 mm from the fovea.
Individual cell type densities were determined as described in Methods.

Supplementary Table 1

Supplementary References

1. Strauss et al. (2021) Center-surround interactions underlie bipolar cell motion sensing in the mouse retina. *bioRxiv* (**Preprint**):1-30.
2. Wässle H, Grünert U, Röhrenbeck J, Boycott BB (1989) Cortical magnification factor and the ganglion cell density of the primate retina. *Nature* **341**:643-646.
