# Supplementary material for: Origins of direction selectivity in the primate retina
Source: Nat Commun. 2022 May 23;13:2862. doi: 10.1038/s41467-022-30405-5 (PMC9126974; doi:10.1038/s41467-022-30405-5)
Supplement: Supplementary file 3 — Reporting Summary [file 41467_2022_30405_MOESM3_ESM.pdf]

nature portfolio

Corresponding author(s): Dennis Dacey

Last updated by author(s): Apr 6, 2022

Reporting Summary

Nature Portfolio wishes to improve the reproducibility of the work that we publish. This form provides structure for consistency and transparency in reporting. For further information on Nature Portfolio policies, see our Editorial Policies and the Editorial Policy Checklist.

Statistics

For all statistical analyses, confirm that the following items are present in the figure legend, table legend, main text, or Methods section.

n/a	Confirmed	
☐	☒	The exact sample size (*n*) for each experimental group/condition, given as a discrete number and unit of measurement
☐	☒	A statement on whether measurements were taken from distinct samples or whether the same sample was measured repeatedly
☐	☒	The statistical test(s) used AND whether they are one- or two-sided<br>*Only common tests should be described solely by name; describe more complex techniques in the Methods section.*
☒	☐	A description of all covariates tested
☐	☒	A description of any assumptions or corrections, such as tests of normality and adjustment for multiple comparisons
☐	☒	A full description of the statistical parameters including central tendency (e.g. means) or other basic estimates (e.g. regression coefficient) AND variation (e.g. standard deviation) or associated estimates of uncertainty (e.g. confidence intervals)
☐	☒	For null hypothesis testing, the test statistic (e.g. *F*, *t*, *r*) with confidence intervals, effect sizes, degrees of freedom and *P* value noted<br>*Give P values as exact values whenever suitable.*
☒	☐	For Bayesian analysis, information on the choice of priors and Markov chain Monte Carlo settings
☒	☐	For hierarchical and complex designs, identification of the appropriate level for tests and full reporting of outcomes
☐	☒	Estimates of effect sizes (e.g. Cohen's *d*, Pearson's *r*), indicating how they were calculated

Our web collection on statistics for biologists contains articles on many of the points above.

Software and code

Policy information about availability of computer code

Data collection	Scan Image Premium (VIDRIO Technology), custom software (Mac OS) for electrophsyiological data acquisition and stimulus generation freely available upon request; NIH FIJI V.2.1 With TraKEM2 plugin, Dragonfly Version 2020 (Object Research Systems).
Data analysis	MATLAB R2020a; Custom MATLAB programs for data analysis freely available upon request. SPSS Version 28.0; Neuron-C

For manuscripts utilizing custom algorithms or software that are central to the research but not yet described in published literature, software must be made available to editors and reviewers. We strongly encourage code deposition in a community repository (e.g. GitHub). See the Nature Portfolio guidelines for submitting code & software for further information.

Data

Policy information about availability of data

All manuscripts must include a data availability statement. This statement should provide the following information, where applicable:

- Accession codes, unique identifiers, or web links for publicly available datasets
- A description of any restrictions on data availability
- For clinical datasets or third party data, please ensure that the statement adheres to our policy

The data that support the findings of this study are available from the corresponding authors upon reasonable request.

Field-specific reporting

Please select the one below that is the best fit for your research. If you are not sure, read the appropriate sections before making your selection.

☒ Life sciences ☐ Behavioural & social sciences ☐ Ecological, evolutionary & environmental sciences

For a reference copy of the document with all sections, see nature.com/documents/nr-reporting-summary-flat.pdf

Life sciences study design

All studies must disclose on these points even when the disclosure is negative.

Sample size	Although no statistical methods were used to predetermine sample size, we used appropriate statistical tests on data at given sample size to test statistical significance. Sample size was determined from the cell count for each experimental condition.
Data exclusions	No data was excluded from this study.
Replication	We replicated our results both within samples (recordings from single cells) and between samples (multiple cell samples). We then used appropriate statistical tests (e.g., Welch's F, Games-Howell post hoc test, paired/and unpaired t-tests) on the data set when the sample size were unequal in order to test statistical significance at given sample size.
Randomization	There were no experimental groups assigned in this study.
Blinding	Blinding was not possible because the same experimenter ran both control and experimental conditions on a given cell sample. However in the case of connectomic reconstruction multiple annotators were used to independently verify "ground truth" electron microscopic reconstructions.

Reporting for specific materials, systems and methods

We require information from authors about some types of materials, experimental systems and methods used in many studies. Here, indicate whether each material, system or method listed is relevant to your study. If you are not sure if a list item applies to your research, read the appropriate section before selecting a response.

Materials & experimental systems

n/a	Involved in the study	
☐	☒	Antibodies
☒	☐	Eukaryotic cell lines
☒	☐	Palaeontology and archaeology
☐	☒	Animals and other organisms
☒	☐	Human research participants
☒	☐	Clinical data
☒	☐	Dual use research of concern

Methods

n/a	Involved in the study	
☒	☐	ChIP-seq
☒	☐	Flow cytometry
☒	☐	MRI-based neuroimaging

Antibodies

Antibodies used	Antibody to Human Melanopsin; personally supplied by Dr. King-Wai Yau (Johns Hopkins University; coauthor on this study); antibodies are available from Dr. Yau upon request.<br>Other commercially available antibodies used:<br>• biotinylated anti-rhodamine (C# BA-0605, Vector Labs, Burlingame, CA<br>• biotinylated goat anti-rabbit (BA-1000; Vector Labs, Burlingame, CA<br>• goat anti-choline acetyltransferase (ChAT) antibodies (AB144P, Millipore)<br>• secondary goat antibodies conjugated to Alexa488 (Invitrogen)
Validation	Antibody to Human Melanopsin was validated in detail: see Liao HW, Ren X, Peterson BB, Marshak DW, Yau KW, Gamlin PD, Dacey DM (2016) Melanopsin-expressing ganglion cells on macaque and human retinas form two morphologically distinct populations. J Comp Neurol 524:2845-2872.

Animals and other organisms

Policy information about studies involving animals; ARRIVE guidelines recommended for reporting animal research

Laboratory animals	Tissues were acquired from Macaque fascicularis, nemestrina and mulatta adults of either sex in the age range 3-15 yrs.
Wild animals	No wild animals were involved in this study.

March 2021

Field-collected samples	This study did not involved samples which are collected from the field.
Ethics oversight	Animal tissues were acquired in accordance with protocols reviewed and approved by the University of Washington Institutional Animal Care and Use Committee.

Note that full information on the approval of the study protocol must also be provided in the manuscript.
